# Measurement equivalence of the SF-36 in the canadian multicentre osteoporosis study

**DOI:** 10.1186/1477-7525-10-29

**Published:** 2012-03-13

**Authors:** Lisa M Lix, Beliz Acan Osman, Jonathan D Adachi, Tanveer Towheed, Wilma Hopman, K Shawn Davison, William D Leslie

**Affiliations:** 1 School of Public Health, University of Saskatchewan, 107 Wiggins Road, Saskatoon, SK, Canada; 2 Health Quality Council, 111 Research Drive, Saskatoon, SK, Canada; 3 Faculty of Health Sciences, McMaster University, 1280 Main St. W, Hamilton, ON, Canada; 4 Department of Community Health and Epidemiology, Carruthers Hall, Queen's University, Kingston, ON, Canada; 5 College of Kinesiology, University of Saskatchewan, 87 Campus Drive, Saskatoon, Canada; 6 Department of Internal Medicine, University of Manitoba, St. Boniface General Hospital, 409 Tache Avenue, Winnipeg, MB, Canada

**Keywords:** SF-36, Health-related quality of life, Psychometrics, Equivalence, Confirmatory factor analysis

## Abstract

**Background:**

Studies that compare health-related quality of life (HRQOL) and other patient-reported outcomes in different populations rest on the assumption that the measure has equivalent psychometric properties across groups. This study examined the measurement equivalence (ME) of the 36-item Medical Outcomes Study Short Form Survey (SF-36), a widely-used measure of HRQOL, by sex and race in a population-based Canadian sample.

**Findings:**

SF-36 data were from the Canadian Multicentre Osteoporosis Study, a prospective cohort study that randomly sampled adult men and women from nine sites across Canada. Confirmatory factor analysis (CFA) techniques were used to test hypotheses about four forms of ME, which are based on equality of the factor loadings, variances, covariances, and intercepts. Analyses were conducted for Caucasian and non-Caucasian females (*n *= 6,539) and males (*n *= 2,884). CFA results revealed that a measurement model with physical and mental health factors provided a good fit to the data. All forms of ME were satisfied for the study groups.

**Conclusions:**

The results suggest that sex and race do not influence the conceptualization of a general measure of HRQOL in the Canadian population.

## Findings

The psychometric properties of health-related quality of life (HRQOL) measures and other patient-reported outcomes are an important consideration when undertaking studies in populations with diverse cultural or racial compositions [1]. The Medical Outcomes Study 36-item Short Form Survey (SF-36) is a well-known HRQOL measure used in many countries. While studies have been undertaken about its reliability and validity in different populations [2], its measurement equivalence (ME) properties have not been well examined. ME evaluations seek to answer the question: "Do individuals from different populations interpret a measure in a conceptually similar manner?" [3]. If ME is not tenable, then researchers cannot validly conclude that differences between groups correspond to true population differences because they will be confounded by measurement artifact. Measurement non-equivalence may exist, in part, because study participants do not interpret questions about their health using the same frame of reference [4,5].

The purpose of this study is to investigate the ME of the SF-36 by sex and race. We focus on its properties in the Canadian population, where normative data for the SF-36 have now been published [6].

## Methods

Study data were from the Canadian Multicentre Osteoporosis Study (CaMos), an ongoing prospective cohort study undertaken to provide national estimates of the prevalence and incidence of osteoporosis and osteoporosis-related fractures. The study population is composed of non-institutionalized men and women residing within a 50-km radius of nine centers across Canada. These geographic areas encompass approximately 40 percent of the national population and include rural and urban residents. A random sample was taken from each site; details of the data collection methodology and participant characteristics have been reported previously [6,7].

The sample consisted of all CaMos respondents for whom baseline data were obtained. Data collection occurred between January 1996 and September 1997 by means of an interviewer-administered questionnaire. Informed consent was obtained from participants and ethical approval was provided by the review boards of each participating center and the coordinating center in Montreal.

The SF-36 encompasses eight domains: physical functioning (PF), role physical (RP), bodily pain (BP), general health (GH), vitality (VT), social functioning (SF), role emotional (RE), and mental health (MH). Each domain is scored on a standardized scale with values ranging from 0 to 100. Higher scores indicate better HRQOL [8]. In previous Canadian research, Cronbach's *α *ranged from 0.76 to 0.93 for the eight domains, with the lowest value for the SF domain [9]. Test-retest reliability has not been reported for the Canadian population, but in other populations a median reliability greater than 0.80 was reported for all but the SF domain, which had a median reliability of 0.76 [10].

Race, age in years, and sex were recorded during the interviews. For race, respondents were initially assigned to Caucasian, Asian, and Other categories. These categories were subsequently collapsed into Caucasian and non-Caucasian.

The data were described using frequencies and means. Hypotheses about ME were initially tested for the following pairs of study groups: (a) Caucasian and non-Caucasian females, (b) Caucasian and non-Caucasian males, (c) Caucasian males and females, and (d) non-Caucasian males and females. Subsequently, we tested ME hypotheses in age-matched groups, in which non-Caucasians were matched with Caucasians using age (in years) as the matching variable. The latter analyses were conducted to adjust for the potential confounding effects of age.

Four forms of ME were investigated using confirmatory factor analysis (CFA) [3,11,12]: configural, weak, strong, and complete. A series of two-group CFA models were fit to the data for each pair of study groups. Weak, strong, and complete invariance was tested in sequence by placing constraints on the parameters (i.e., factor loadings, intercepts, and error variances) of the configural invariance model [3,11]. Configural invariance, the simplest form of ME, is satisfied if a defined factor structure is a good fit to the data for both groups. It was evaluated using absolute and incremental goodness-of-fit statistics and published cut-off criteria [13-15]. The statistics included the model *χ*^2^, root mean square error of approximation (RMSEA) and its 90% confidence interval (CI), root mean squared residual (SRMR), comparative fit index (CFI) and non-normed fit index (NNFI). Model modification indices were calculated for the configural invariance model to guide decisions about its specification. These indices measure the predicted change in the *χ*^2 ^statistic if a parameter is added or removed from the model and re-estimated.

A test of weak invariance assesses whether the factor loadings are the same for the groups. When weak invariance is satisfied, the latent variables are being measured in the same way for the groups. A test of strong invariance is used to assess whether the factor loadings and latent variable intercepts (i.e., means) are the same for the groups. If strong invariance does not hold then it is not valid to make group comparisons on the domain means. Complete invariance holds if the factor loadings, intercepts, and error variances are equivalent for the groups [11]. A LR statistic based on the difference in *χ*^2 ^values for unconstrained and constrained models (i.e., Δ *χ*^2^), was used to test weak, strong, and complete invariance. The difference in CFI values for nested models (i.e., ΔCFI) was also used to assess invariance because the LR statistic is sensitive to sample size. An absolute value of ΔCFI less than or equal to 0.01 indicates the null hypothesis of invariance should not be rejected, while an absolute value greater than or equal to 0.02 indicates a likely difference in fit between constrained and unconstrained models [16]. ΔCFI was given more weight than the LR test when there was disagreement between the two statistics.

Robust maximum likelihood was used to estimate model parameters because the data exhibited a multivariate non-normal distribution [17]. Accordingly, Satorra-Bentler (SB)-scaled *χ*^2 ^statistics, which correct for non-normality using RML were adopted [18]. Analyses were conducted using LISREL 8.80 [19].

## Results

Data for 9,423 CaMos participants (Table 1) were included in the analysis. Two-thirds of participants were female. The majority (94.9%) was Caucasian and this percentage was similar for males and females. Average scores for each of the SF-36 domains (Table 2) revealed that males tended to have higher HRQOL than females. For females, scores for Caucasians were often lower than those for non-Caucasians. For males, this was not always the case.

**Table 1:** Distribution of the CaMos cohort by sex, age, and race

Age (years)	Female						Male					
	
	Caucasian		Asian		Other		Caucasian		Asian		Other	
	*n*	*%*	*n*	*%*	*n*	*%*	*n*	*%*	*n*	*%*	*n*	*%*

25-49	902	14.1	30	22.1	41	29.3	639	23.8	28	26.9	30	30.9

50-59	1,274	20.3	40	20.4	38	27.1	554	20.7	18	17.3	30	30.9

60-69	1,963	31.3	42	30.9	39	27.9	711	26.5	25	24.0	23	23.7

70+	2,124	33.9	24	17.7	22	15.7	779	29.0	33	31.7	14	14.4

Total	6,263	100.0	136	100.0	140	100.0	2683	100.0	104	100.0	97	100.0

**Table 2:** Means and standard deviations (SDs) for the SF-36 domains

Domain	Race	Female	Male
Physical functioning	Caucasian	73.49 (25.32)	81.33 (22.27)
	
	Non-Caucasian	79.69 (21.18)	82.17 (21.57)
	
	All Groups	73.75 (25.19)	81.39 (22.22)

Role physical	Caucasian	74.08 (38.16)	81.50 (33.28)
	
	Non-Caucasian	79.62 (35.85)	81.84 (33.72)
	
	All Groups	74.31 (38.08)	81.53 (33.31)

Bodily pain	Caucasian	70.82 (24.61)	76.79 (22.46)
	
	Non-Caucasian	76.00 (24.18)	79.41 (24.25)
	
	All Groups	71.04 (24.61)	76.97 (22.59)

General health	Caucasian	73.92 (19.04)	74.69 (17.90)
	
	Non-Caucasian	70.95 (19.37)	73.37 (19.19)
	
	All Groups	73.80 (19.06)	74.59 (17.99)

Vitality	Caucasian	62.63 (19.87)	67.80 (17.83)
	
	Non-Caucasian	64.74 (19.42)	68.93 (17.23)
	
	All Groups	62.71 (19.85)	67.88 (17.78)

Social functioning	Caucasian	85.49 (21.37)	89.03 (18.71)
	
	Non-Caucasian	84.60 (21.42)	83.21 (22.21)
	
	All Groups	85.46 (21.37)	88.62 (19.03)

Role emotional	Caucasian	83.56 (32.07)	87.90 (27.82)
	
	Non-Caucasian	82.61 (34.75)	82.26 (33.83)
	
	All Groups	83.52 (32.19)	87.50 (28.31)

Mental health	Caucasian	77.78 (15.56)	81.09 (13.89)
	
	Non-Caucasian	78.23 (16.91)	80.89 (14.89)
	
	All Groups	77.80 (15.62)	81.08 (13.95)

The non-Caucasian group includes Asian and Other groups

**Figure 1:**
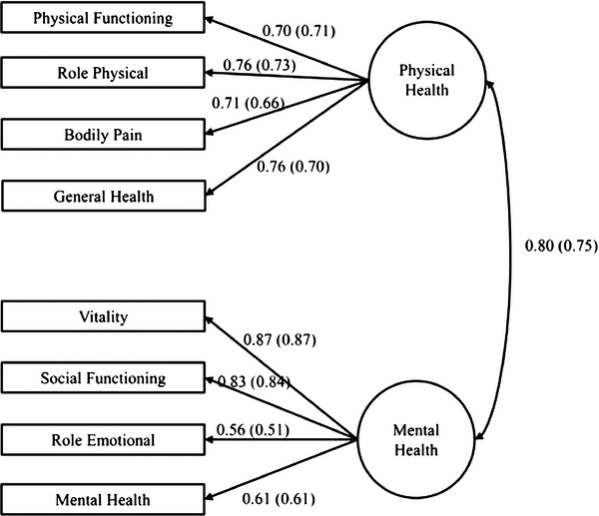
**Initial configural invariance model for the SF-36**. The circles represent the SF-36 physical and mental health latent factors, the rectangles represent measured indicators (i.e., the domains), the lines connecting latent factors to indicators are factor loadings, and the curve connecting the two latent factors represents covariation. Numbers provided are standardized values for female Caucasians and male Caucasians (in parentheses).

The initial configural invariance model (Figure 1) was fit to the data for each study group. This model was selected based on previous research that supports a two-factor model with four domains each measuring physical and mental health latent variables [10]. Based on the *χ*^2 ^statistic, RMSEA, and SRMR (Table 3), this model did not provide a good fit to the data. Model modification indices suggested that substantial improvement in fit could be obtained by including covariances among the residual errors of the SF-36 domains for RP and GH, VT and SF, and RP and RE. With these modifications (Table 3), all goodness-of-fit statistics indicated a well-fitting model.

**Table 3:** Fit statistics for initial and modified configural invariance model for the SF-36

Study group	Model	SB *χ*^2^	RMSEA (90% CI)	SRMR	CFI	NNFI
Female, Caucasian	Initial	1584.95*	0.12 (0.11, 0.12)	0.06	0.96	0.94
	
	Modified	976.36*	0.10 (0.09, 0.10)	0.05	0.97	0.96

Male, Caucasian	Initial	718.18*	0.12 (0.11, 0.13)	0.07	0.95	0.92
	
	Modified	404.36*	0.10 (0.09, 0.10)	0.05	0.97	0.95

Female, non-Caucasian	Initial	81.58*	0.11 (0.09, 0.14)	0.06	0.96	0.95
	
	Modified	45.64*	0.08 (0.06, 0.11)	0.04	0.98	0.97

Male, non-Caucasian	Initial	68.23*	0.11 (0.09, 0.14)	0.07	0.95	0.93
	
	Modified	33.35*	0.07 (0.04, 0.11)	0.05	0.98	0.97

SB = Satorra-Bentler; RMSEA = root mean square error of approximate; CI = confidence interval; CFI = comparative fit index; NNFI = non-normed fit index; Initial model, which is defined in Figure 1, has 19 degrees of freedom and the modified model has 16 degrees of freedom; * denotes a SB *χ*^2 ^statistic that is statistically significant at *α *= .05

The hypothesis of weak invariance was tested for this measurement model (Table 4). It was retained for all pairs of subgroups based on the ΔCFI, although the LR statistic was statistically significant for Caucasian and non-Caucasian females. The null hypothesis of strong invariance was retained for all pairs of study groups based on the ΔCFI statistics. Finally, the hypothesis of complete invariance was retained for all pairs of study groups according to ΔCFI statistics. Subsequent analyses for the age-matched study groups resulted in the same conclusions about all ME hypotheses.

**Table 4:** Tests of measurement equivalence for the SF-36

Equivalence Hypothesis	SB *χ*^2^	*df*	Δ SB *χ*^2^	Δ*df*	CFI	ΔCFI
	Caucasian and non-Caucasian females					

Configural	1067.88	32	-	-	0.97	-

Weak	1077.48	38	22.58*	6	0.97	0.00

Strong	1212.23	46	82.05*	8	0.97	0.00

Complete	1174.84	60	21.67	14	0.97	0.00

	Caucasian and non-Caucasian males					

Configural	486.87	32	-	-	0.97	-

Weak	500.21	38	10.80	6	0.97	0.00

Strong	561.17	46	41.58*	8	0.96	0.01

Complete	501.75	66	28.40*	14	0.97	0.01

	Caucasian females and males					

Configural	1356.11	32	-	-	0.97	-

Weak	1355.13	38	6.74	6	0.97	0.00

Strong	1693.35	46	364.97*	8	0.97	0.00

Complete	1966.86	60	242.87*	14	0.96	0.01

	non-Caucasian females and males					

Configural	79.49	32	-	-	0.98	-

Weak	83.33	38	4.69	6	0.98	0.00

Strong	99.81	46	16.23*	8	0.98	0.00

Complete	106.53	60	10.84	14	0.98	0.00

SB = Satorra-Bentler; * denotes a SB *χ*^2 ^statistic that is statistically significant at *α *= .05

## Discussion

This study investigated the psychometric equivalence of the SF-36 by sex and race in a population-based cohort that represents a large proportion of the Canadian population. These stratification variables were selected because previous research indicates they are associated with differences in the conceptualization of HRQOL and other patient-reported outcomes [2]. All forms of ME were supported in each of the four analyses.

This study adopted stringent criteria for establishing ME of the SF-36. While configural and weak invariance are usually tested, Gregorich [20] notes that strong and complete invariance are less frequently considered, despite the fact that equality of factor loadings, intercepts, and error variances is critical to making valid group comparisons [21]. Vandenberg and Lance [11] found that weak invariance was investigated in 99% of studies but strong invariance was tested in only about 12% of studies. However, this research also has some limitations. ME was investigated for a single measure; other measures of HRQOL might not be psychometrically equivalent. Other stratification variables may have been considered in the ME models, such as education [22]. However, further stratification of the data would have resulted in sample sizes too small to result in valid tests of the study hypotheses. The initial factor structure selected for the SF-36 domains did not provide a good fit to the data. It was modified to allow for correlation among the residual errors of selected domains. While this model was consistent with previous research [23], it may not be consistent with the measurement model adopted in other studies. Finally, only a single statistical method, CFA, was used to test ME. Item response theory has also been proposed for evaluating equivalence and these approaches may not concur [24].

Establishing ME across populations is a prerequisite for conducting valid tests of hypotheses about equality of group means or variances. The findings of this study suggest that sex and race do not influence the conceptualization of a general measure of HRQOL in the Canadian population.

## Abbreviations

BP: Bodily pain; CaMos: Canadian Multicentre Osteoporosis Study; CFA: Confirmatory factor analysis; CFI: Comparative fit index; CI: Confidence interval; GH: General health; HRQOL: Health-related quality of life; ME: Measurement equivalence; MH: Mental health; NNFI: Non-normed fit index; PF: Physical functioning; RE: Role emotional; RMSEA: Root mean square error of approximation; SRMR: Root mean squared residual; RP: Role physical; SB: Satorra-Bentler; SF: Social functioning; SF-36: 36-item Medical Outcomes Study Short Form Survey; VT: Vitality.

## Competing interests

The authors declare that they have no competing interests

## Authors' contributions

LML planned the study and analyses and drafted the manuscript. BAO conducted the analyses and drafted the manuscript. JA, TT, SD, WH, and WDL participated in planning the study and facilitating data access. All authors read and approved the final manuscript.
